# Uncovering the Source of Patrimonial Voting: Evidence from Swedish Twin Pairs

**DOI:** 10.1007/s11109-020-09669-4

**Published:** 2021-01-30

**Authors:** Rafael Ahlskog, Anton Brännlund

**Affiliations:** Department of Government, Uppsala University, Gamla Torget 6, Uppsala, Sweden

**Keywords:** Patrimonial voting, Discordant twin design, Wealth, Political preferences

## Abstract

The boom in wealth inequality seen in recent decades has generated a steep rise in scholarly interest in both the drivers and the consequences of the wealth gap. In political science, a pertinent question regards the political behavior across the wealth spectrum. A common argument is that the wealthy practice patrimonial voting, i.e. voting for right-wing parties to maximize returns on their assets. While this pattern is descriptively well documented, it is less certain to what extent this reflects an actual causal relationship between wealth and political preferences. In this study, we provide new evidence by exploiting wealth variation within identical twin pairs. Our findings suggest that while more wealth is descriptively connected to more support for right-wing parties, the causal impact of wealth on policy preferences is likely highly overstated. For several relevant policy areas these effects may not exist at all. Furthermore, the bias in naive observational estimates seems to be mainly driven by environmental familial confounders shared within twin pairs, rather than genetic confounding.

**Supplementary information:**

The online version of this article (10.1007/s11109-020-09669-4) contains supplementary material, which is available to authorized users.

## Introduction

Where do political preferences come from? A popular view is that much of our attitudes are shaped by economic motivations and that policy preferences change with the size of our pocketbooks (Margalit 2019). There is now a growing scholarly interest in wealth as the world has seen a massive, but unevenly distributed, increase in asset wealth over recent decades. Fuelled by globalisation and financial deregulation, the current growth in wealth is outpacing both global gross domestic product (GDP) and population growth. National wealth-to-income ratios are now approaching levels not seen since the outbreak of World War I (Piketty and Goldhammer 2014).

The wealth gap divides countries but also generations. While the baby boom generation has been described as the wealthiest generation of all time, their grandchildren are to a much greater extent struggling with debt to afford necessities such as education and housing. An increased level of conflict between the have and have nots is to be expected if economic interests determine preferences over important policies such as redistribution.

Being wealthy is associated with better health and life-satisfaction (Lindqvist et al. 2010; Hajat et al. 2010), while falling behind on the economic ladder is associated with depression and psychological stress (Patel et al. 2018; Silva-Ribeiro et al. 2017). Thus, it is not surprising that the scholarly interest in inequality has revived the political discussion about wealth redistribution. Hundreds of thousands of fortunate millionaires are created each year and their political influence is growing—issues raised by the wealthy are systematically more likely to be addressed by elected officials (Gilens and Page 2014; Powell and Grimmer 2016).

This raises intriguing questions about the observed differences in attitudes between wealthy and poor citizens and effects on preference formation of increases in wealth. This is important, especially since the growing wealth gap is driven not only by market forces but also by deliberate public policy, in that less financial regulation and lower capital taxes disproportionally benefit the already wealthy (Tanndal and Waldenström 2017).

The relationship between self-interest and political preferences has been at the centre of political economy writ large since its inception, ranging from Marx and Smith to modern work in public choice. The well and the less well-off are assumed to hold a different set of policy preferences, or even political identities, due to their relative position along the wealth distribution. More generally, accounts of political preference formation that rely on material self-interest imply that changing economic circumstances should lead to changes in relevant policy positions.

A recent wave of studies has estimated a positive relationship between holding financial assets and voting for right-wing parties. Students of patrimonial voting argue that wealthy citizens, especially owners of high-risk assets are more supportive of conservative parties since they expect to benefit from free-market politics (Lewis-Beck et al. 2013; Foucault et al. 2013; Nadeau et al. 2010, 2011). Scholars such as Ansell (2014) argue on the other hand that wealth makes citizens hostile against redistribution because private assets reduce the need for welfare spending. However, it is less clear whether this association actually reflects a true causal signal such that an increase in asset wealth leads to a corresponding shift in political preferences.

Most importantly, there are a number of confounding factors that preclude causal conclusions. For example, people inherit both economic resources and political attitudes from their parents, as well as genetic proclivities for both (Piketty 2011; Ohlsson et al. 2019; Benjamin et al. 2012; Alford et al. 2005; Hatemi et al. 2019). This means that rearing environment and genetic overlap are important underlying variables to consider—factors that are often overlooked. Second, there might be issues with reverse causation. A growing body of research argues that economic outcomes could themselves be functions of political beliefs. For instance, studies show that citizens engage in different types of consumption and investment patterns, or interpret changes in the economic context differently, depending on their political identity (Evans and Andersen 2006; Ramirez and Erickson 2013; Key and Donovan 2017; Hong and Kostovetsky 2012; Kaustia and Torstila 2010). While there is an emerging literature that attempts to circumvent these issues with quasi-experimental designs, such as analyzing large lottery winnings (Doherty et al. 2006; Peterson 2016; Powdthavee and Andrew 2014), these types of wealth shocks are exceedingly rare and often disproportionally large, and therefore likely of little practical importance to policy.

Our aim with this study is to provide new evidence on the possible causal connection between wealth and political preferences using variation from Swedish middle-aged twin pairs, and assess to what extent this association might be driven by genetic and familial environmental confounders. This sample represents a group of people who are past their impressionable years, and who are also exposed to differences in wealth in a range that is vastly more common than, for example, large lottery winnings. Using discordant twin methods allows us to isolate the effect of wealth changes later in life from shared early life experiences and genetic effects. Leveraging differences between identical and fraternal twins with bivariate decomposition techniques further make it possible to disentangle to what extent genetic factors as well as shared environmental factors, respectively, drive the naive association between wealth and political preferences. We find that the impacts of wealth on political preferences are, at least in this more narrow sense, probably highly overstated. We also find that the sources of the bias in naive observational estimates appear to be mostly of environmental nature, where environmental influences shared within twin pairs account for a substantial proportion of the covariance.

## Previous Research and Hypotheses

A standard assumption within the school of public choice is that political views are self-serving: we will tend toward policy positions that are consistent with our self-interest. Other schools of thought contrast this argument by illustrating the lasting impact of early life experiences. In fact, already at the time of our birth we share certain genetic predispositions for political traits with our siblings, inherited from our parents (Alford et al. 2005; Hatemi et al. 2019). Later, and throughout childhood, we become even more similar to our siblings as we adapt and learn in shared social environments (Healy and Malhotra 2013; Jennings et al. 2009; Jennings and Niemi 1968). When we reach the age span between 18 and 25, also known as the “impressionable years”, we develop attitudes that we may also share with others in our generation, given that we respond to the wider economic and political environment (Jennings and Niemi 1981). In short, growing up in different political and economic contexts will greatly affect how we feel about policy issues later in life (Alesina 2007; Giuliano and Spilimbergo 2014).

Political preferences, once formed, are said to remain sticky, which would appear to leave little room for current economic conditions to have any dramatic effects on political behavior. Simply put: how we behave as adults is partly a product of both genetic inheritance and socialization in early years and this political foundation is said to impact all types of behavior later in life. Moreover, the “Michigan model” states that psychological attachment raises a perceptual screen such that individuals tend to see what is favorable to their political bias (Campbell 1960). In line with this thesis, other studies confirm that political beliefs affect everything from consumption and investment behavior (Key and Donovan 2017; Hong and Kostovetsky 2012; Kaustia and Torstila 2010) to how we selects our spouses (Iyengar et al. 2018). These findings raise some doubts about treating humans as primarily economic voters (Evans and Andersen 2006; Ramirez and Erickson 2013). However, throughout our lives, we encounter situations where our political identity comes into conflict with our self-interest. Having a left-wing identity is for instance not an obstacle to achieving financial success given that when asset prices rise, all owners benefit regardless of political background.

### Hypotheses

Since the financial crisis, asset prices in both the US and Europe have risen to new record levels and the theory of patrimonial voting suggests that higher asset prices benefit those with larger asset holdings. By holding assets, especially those with a high-risk profile, the incentives to support parties and candidates who endorse low taxation and financial deregulation increase since asset returns are expected to rise under such policies. Examples of risky patrimony are private equity, stocks or other types of financial products. These asset classes require a more active management strategy compared to less risky investments such as real estate. Thus, owners of high-risk portfolios will strive to maximize their returns and this makes them less likely to support intervention in the market compared to owners of low-risk assets (Nadeau et al. 2010, 2011). This effect is further said to increase with the size of the asset portfolio (Foucault et al. 2013).

The relationship between holding risky assets and political preferences has empirical support from multiple countries (Foucault et al. 2013; Stubager et al. 2013; Lewis-Beck et al. 2013), but is also said to vary, both with the institutional setting and the political landscape (Quinlan and Okolikj 2019; Hellwig and McAllister 2019). Evidence from Sweden suggests, for instance, that highly valued real estate produces similar effects as well and that political preferences are mainly driven by total asset value rather than risk-profile (Persson and Martinsson 2018).^1^ Hence, in the Swedish context we hypothesize that:

#### H1a

Wealthier individuals are more likely to support free-market policies.

Today, the wealthiest hold most of the world’s financial assets while the wealth of the average citizen comes in the form of real assets—namely housing (Wind et al. 2017; Bertaut et al. 2002). An implication of this is that a particularly salient—and also direct—type of pocketbook mechanism between asset holdings and political attitudes should be between real asset holdings and attitudes about real-estate taxation. This is especially true in the context and time period where we have data. Sweden is similar to many other western nations in that housing wealth is the dominant wealth type for the typical household. Property taxes were also highly visible to homeowners between 1985 and 2008 since owners were liable to pay a fee based on property values once a year. Individuals with otherwise very similar characteristics were during this period exposed to varying financial pressures based on trends in neighborhood house prices. It therefore makes sense to treat property taxes as a most likely case in this population.

#### H1b

Individuals with larger real wealth are more likely to oppose real-estate taxation.

Certain wealth disparities can also generate conflicts over social policy. When we are young, we depend on welfare services such as education. As adults, some come to depend on unemployment benefits while becoming old is associated with a higher demand for subsidized health care and public pension. This is why age is a suggested driver behind social policy preferences (Busemeyer et al. 2015). Asset wealth on the other hand, is assumed to push us away from this traditional path.

Becoming wealthy is said to decrease demand for welfare services in that private wealth can be substituted for social spending (Ansell 2014). However, wealth may not simply transform us into strict fiscal conservatives but rather change our priorities. Put simply, as we become better-off we also come to benefit from government polices such as mortgage deductions, tax-breaks or subsides on certain services and businesses. Even more importantly, with a bigger stake in the market, we come to demand government protection from falling asset prices (Chwieroth and Walter 2019). However, to capture the essence of this effect, it is important to separate financial from non-financial wealth given that for homeowners, these real holdings are of substantially less help in times of financial distress, since liquidating this wealth comes with a host of problems of its own. Limited access to financial wealth should increase the demand for social spending given that liquidity constrained individuals will have a harder time to cope in case of unemployment. Hence, we hypothesize:

#### H2

Individuals with more financial assets are less likely to support social spending.

Finally, how we behave on election day can be influenced by how much wealth we have accumulated. The theory of pocketbook voting predicts self-serving behavior at the ballot box in that we aim to maximize our private finances through our vote choices. As wealthy we follow patrimonial voting patterns by supporting fiscally conservative parties (Lewis-Beck et al. 2013; Foucault et al. 2013; Persson and Martinsson 2018; Quinlan and Okolikj 2019), and vice versa. The source of this pattern lies in the conflict on how resources should be distributed over society given that party cleavages have traditionally been centred around economic policy. Furthermore, in a Scandinavian context the left–right continuum is traditionally almost exclusively perceived as an indicator of economic and redistributive policy. Right-wing parties are from this perspective more likely to pursue polices that benefit the wealthy financially.

#### H3

Wealthier individuals are more likely to support right-wing parties.

While previous observational studies have found consistent relationships between economic resources and political preferences, they typically suffer from several types of endogeneity problems that make causal claims difficult even when using a rich set of controls. One particularly salient problem has recently been given more attention, and arises if both traits are genetically heritable and also share some degree of genetic etiology. This leads to a correlation between the two traits that is due to being influenced by shared genetics, rather than one causing the other. The problem is often called genetic confounding, and is very difficult to control for in observational studies unless some type of genetically informative data is used.

Wealth and ideology have both previously been shown to have a moderate level of heritability. For example, Alford et al. (2005) and Hatemi et al. (2019) show that some degree of similarity in political preferences between parents and children are due to genetic transmission, while Benjamin et al. (2012) document heritability for both life income and wealth. Since both the independent and dependent variables in this case are heritable, any observational study that does not use genetically informative data runs the risk of being confounded by shared genetics.

Credible causal evidence for a wealth effect on political preferences comes mainly from lottery studies. However, lottery winnings often represent an extreme tail-end economic shock at the individual level, and the question remains whether changes in wealth within a more conventional range of variation has the same effect. Additionally, lottery players may differ systematically from non-players in a way that makes generalizations of these results difficult. For example, they may be less risk-averse, but may also be more interested in financial wealth than others, possibly making them more likely to also be susceptible to rational choice-type mechanisms in the first place.

In this paper we will attempt to test the above outlined hypotheses on the effects of wealth on political preferences. The unique contribution of this study is three-fold. First, we utilize a discordant twin design that eliminates the problem of genetic and shared environmental confounding. Second, using twin data also allows us to directly assess the degree to which naive observational estimates of these relationships may be biased by genetic and environmental confounders. Third, we observe wealth counterfactuals within a conventional range of variation which greatly improves the external validity, and possibly the policy relevance, of the results.

## Methods

This study utilizes a two-pronged twin data approach. First, a discordant MZ twin design will illuminate whether correlations between wealth and the theoretically relevant political orientations are robust to genetic and common environmental confounding. Second, bivariate ACE models are used to investigate the extent and relative magnitude of such confounding. Both of these approaches are described below.

### Discordant MZ Twin Design

The discordant twin design departs from a pseudo-experiment that nature provides us with: identical twinning. Identical (or monozygotic, MZ) twins share (minus extremely rare mutations) all of their DNA. Comparing identical twins with each other thus allows us to control for genetic confounding since genotype is held constant. They will also, generally speaking, share the same home environment, meaning that unmeasured environmental confounders shared by both twins are also controlled for, such as parenting and shared social network effects. The design is not precisely an experiment in the true sense of the word, but it allows us to get much closer to the causal variation since any confounders of either a genetic or shared environmental nature are held constant (Vitaro et al. 2009).

Discordant twin designs have been used productively in both medical and psychological research for several decades, and is a prime examle of how genetically informed research designs can be used to robustly illuminate questions of a purely environmental nature (Pike et al. 1996). The approach has also recently made headway in political science, primarily in the study of educational effects (e.g. Oskarsson et al. 2016; Dinesen et al. 2016; Weinschenk and Dawes 2019 and Robinson 2020).

In essence, the discordant twin design uses within-pair comparisons rather than cross-pair comparisons. If wealth has an independent effect on ideology, as opposed to wealth and ideology sharing the same genetic or environmental etiology, the wealthier twin within a pair should also, on average, have different political preferences. Technically speaking, this amounts to simply running regressions with fixed effects at the twin-pair level. Even within identical twin pairs, however, there can still be confounders of the relationship of interest—environmental variables not shared within pairs could be causally prior to both the dependent and the independent variable. One generally therefore includes some suitable controls for these non-shared variables (these are outlined below), resulting in the following model:

$$\begin{aligned} y_{i} = a + b x_{i} +\sum \gamma _{j}d_j + \sum c_{k}z_{ki} + e_{i} \end{aligned}$$

where $x_{i}$ is the level of wealth of person *i*, $\gamma _{j}$ is the fixed effect for twin pair *j* and $z_{ki}$ is the value for control variable *k* for individual *i*.

Since the model includes observations for both twins in a pair, standard errors are also clustered at the twin pair level. Naive models (i.e. without twin pair fixed effects) are reported as points of comparison.

Causal precision, when using this method, does come at a price however. In particular, the amount of variation within twin pairs in the independent variable is going to be lower than the aggregate level of variation across the sample. This means that within-pair models are bound to lose some amount of statistical precision. The feasibility of the method therefore depends on there being enough within-pair variation to exploit. We delve into this issue in more detail in the descriptives section below.

### Bivariate ACE Decomposition

While the results from the discordant twin models can support or refute the hypotheses we intend to test, they do not give any answer as to *why* an effect may change when controlling for shared twin factors. Broadly speaking, a change in effect size could result from either confounding environmental factors that the twins share, or from genetic confounding (wealth acquisition and political attitudes may both stem from some underlying genetically influenced trait). Knowing the extent to which these two sources of confounding affect the results is important for future research, since the respective types of confounding have different implications for what type of additional data would be needed in observational research to handle.

To map out the *sources* of any unmeasured (genetic or environmental) confounding, variation among same-sex fraternal (dizygotic, or DZ) twins can also be leveraged. The identifying variation lies in the differential genetic overlap: while identical twins share 100% of their genetic material, fraternal twins share on average 50%. At the same time, both twin pairs will also share their family environment. This means that a larger degree of similarity for any given trait among MZ twins as opposed to DZ twins can be attributed to genetic factors, while the remaining degree of similarity when this has been accounted for must be due to shared environmental factors. Typically, such variance decomposition models partition the total variance in a trait into three sources: an additive genetic component (A), shared environmental sources (C) and non-shared environmental sources (E).

With two traits (such as wealth and ideology), variance decomposition models allow us to map whether a correlation between the traits can be attributed to overlapping genetic causes (this is called genetic correlation) or overlapping environmental causes. Once again, it’s intuitive to see that if the correlation between trait 1 in twin 1 and trait 2 in twin 2 is higher among identical twins, the two traits must share a common genetic etiology.

We use bivariate decompositions to obtain genetic correlations as well as environmental correlations (i.e. to what extent a correlation between two traits can be attributed to the same shared environmental influences). In so doing, estimates for the univariate heritability of each trait are also derived and presented.^2^

### Data and Variables

This study uses data from the extensive Swedish twin registry as well as other register data sources.^3^ The twin registry contains, among other things, information about zygosity, as well as a range of surveys relevant to our hypotheses.

For the dependent variables, we have used data from the SALTY survey, administered in 2009/2010 to twins born between 1943 and 1958 (and thus of ages 51–66 at the time of the survey). This survey contains a battery of 34 questions on opinions about specific political issues, as well as direct questions on left–right placement and party choices. Furthermore, this sample is of particular interest since a sound labor market between 1960 and 1980 allowed many in the baby boom cohorts to invest in housing which later made them into “accidental millionaires” as housing prices increased.

H1a regards the effect of wealth on support for free-market policies. We have chosen to operationalize this as an additive index containing the following issue items: support for lower taxes (+), keep property taxes (−), privatize public enterprises (+), decrease the impact of financial markets (−) and give companies more freedom (+). The first two items are self-explanatory in that higher taxes impliy smaller return on any given asset portfolio. Regarding the last two items, we argue that these indicators capture attitudes on the regulation of markets and where less regulatios is assumed to be good for asset prices. One of the subcomponents (keep property taxes), measured as degree of support for keeping property taxes on a scale from 1 to 5, is also used as a pure pocket book indicator to evaluate H1b.

H2 on the other hand regards attitudes toward redistributive social policies. This dimension is operationalized in a similar way using the following issue items: decrease the public sector (−), decrease social insurance reimbursements (−), decrease income inequality (+), privatize healthcare (−), keep price ceilings in child care (+) and support free schools^4^ (−).

H3, finally, is about the general political spectrum rather than specific issue questions. For this, we have two variables: first, self-reported left–right placement. Second, self-reported party choice in the previous (2006) parliamentary elections. This has been recoded as an ordinal scale where parties are placed from left (0) to right (7).^5^

All dependent variables were then rescaled to range from 0 to 1 using min and max observed values as endpoints.^6^

Data for the main independent variable (wealth) was obtained from the Wealth registry (Förmögenhets-registret). The Wealth registry existed from 1999 to 2007 (when the wealth tax in Sweden was abolished) and contains both gross and net wealth, as well as gross wealth divided into real and financial wealth. As our independent variable of interest, we have opted for gross wealth, since the mechanisms described in the theory section all depend on the value of assets, rather than the balance of assets to debt. For example, if one decides on a preference for real estate taxation, it is the value of the real estate asset, rather than whether one still has debt left to pay off, that is the relevant factor.

To obtain individual level data less susceptible to year-by-year variation (for example due to booms or busts on the financial or housing markets), the average gross wealth over all nine years is used, expressed as million SEK. The resulting variable was also trimmed at the 99:th percentile to exclude a small number of extreme outliers (some observations were more than 100 standard deviations from the mean).^7^ A robustness check using a hyperbolic sine transformation of wealth is also reported in Appendix A, to address concerns of non-linear effects of wealth.

As stated above, even within identical twin-pairs there can be confounding variables: these will generally be of the type that are usually labeled non-shared environmental variables—environmental factors that differ between the two twins and are causally prior to both individual wealth and political attitudes. Controls are also included to get a reference point on how much bias can be removed with control variables alone.

A major possible confounding factor in this context is work income. It is therefore included as a control. Data on working income was taken from the LISA databases. An average was taken of the annual work income for the 10 years prior to answering the questionnaire. As with wealth, extreme outliers were removed by trimming at the 99th percentile.

A common control in studies investigating the relationship between wealth and political preferences is occupational category. We argue that this control is problematic, since it will also pick up all of the covariation between wealth and political preferences that is actually *mediated*, rather than confounded, by occupation. For example, it is not difficult to imagine that once a certain wealth threshold is passed, some people choose to opt out of employment altogether. Meanwhile, the number of occupations that cause sizable changes in wealth (as opposed to work income) are vanishingly few. Still, it is not inconceivable that there is some residual confounding picked up by occupational category, over and above that of income, and occupation may also absorb enough variation in political preferences to increase the precision of the models. For these reasons, we will present models both with and without occupational category as a control. 

Furthermore, education conceivably influences both processes of wealth acquisition as well as political preferences, meaning that within-pair correlations between the two could be biased if education was left uncontrolled for. It is also unlikely to be a mediator, and is therefore included in the main specifications. Data on years of education was calculated based on highest achieved level of education in the LISA databases. The number of non-adult children are also included as controls, as is biological sex for naive models (within identical twin pairs, biological sex is invariant). Finally, models are included where the *change* in wealth in the years preceding the survey is controlled.

**Table 1:** Descriptive statistics

Variables	N	Mean	SD	Min	Max
MZ pairs					
Free market preferences	2255	0.474	0.173	0	0.938
Redistributive preferences	2239	0.591	0.175	0.0455	1
Left–right orientation	2368	0.507	0.268	0	1
Party choice (L–R)	2140	0.468	0.326	0	1
Property tax support	2325	0.392	0.312	0	1
Wealth, MKr	2303	0.957	0.893	0	4.340
Financial wealth, MKr	2299	0.195	0.268	0	1.554
Real wealth, MKr	2303	0.709	0.682	0	3.178
Work income	2309	0.354	0.185	0	0.994
Education years	2368	12.08	2.591	7	19
Children under 18	2367	0.0824	0.341	0	3
DZ pairs					
Free market preferences	1725	0.456	0.174	0	1
Redistributive preferences	1708	0.602	0.172	0	1
Left–right orientation	1833	0.466	0.275	0	1
Party choice (L–R)	1661	0.420	0.320	0	1
Property tax support	1790	0.420	0.310	0	1
Wealth, MKr	1779	0.862	0.800	0	4.308
Financial wealth, MKr	1789	0.183	0.255	0	1.641
Real wealth, MKr	1779	0.650	0.639	0	3.151
Work income	1794	0.343	0.173	0	0.978
Education years	1832	11.79	2.683	7	19
Children under 18	1831	0.0732	0.314	0	3

### Descriptives

The descriptives for all variables, for the two core samples (identical twins for the main analyses and fraternal twins for the bivariate ACE decompositions) can be found in Table 1. As we can see, the average wealth is slightly less than one million Swedish krona, with roughly three quarters of the wealth being in the form of real assets.^8^

Meanwhile, Table 2 contrasts with the within-pair variation in wealth among the identical twins. Since this is the identifying variation, it is important that there is enough of it for a discordant analysis to make sense. Several indicators can be compared to get a sense of the within-pair distribution. First of all, the absolute within-pair differences in the different wealth categories are informative. We can see that these are roughly .58 million SEK for general wealth, .16 million for financial wealth and half a million for real wealth. Considering that this is generally more than half of the averages for each category across the sample (from Table  1), this should leave more than enough variation to work with. A second thing to note is the comparison of the within-pair standard deviation to the pooled (across sample) standard deviation. We can see that, as expected, the within-pair standard deviations are lower (about one third for each wealth type), but not so low as to pose a problem for the identification strategy.

**Table 2:** Within-pair variation, MZ

	Abs. diff. mean	Abs. diff. min/max	Within SD	Across SD
Wealth, MKr	.58	0/4.16	.29	.89
Financial wealth, MKr	.16	0/1.57	.08	.27
Real wealth, MKr	.49	0/2.87	.24	.68
$\varDelta$ Wealth, MKr	.94	0/71.67	.47	2.86
$\varDelta$ Fin. wealth, MKr	.39	0/44.82	.18	.91
$\varDelta$ Real wealth, MKr	.80	0/68.40	.40	2.61

These figures are also informative for the question of where within-pair differences in wealth stem from. We can see, for example, that the within-pair differences are of the same type as across-pair differences: mainly in real wealth. This can be seen more clearly when looking at within-pair differences in how the asset values have developed over time (from the start of the measurement period for wealth to the end, i.e. 1999–2007). Here we can see that there is substantial differences in volatility over time: the average difference in the change in gross wealth within pairs is close to one million SEK. This volatility is also captured mainly by differences in the development of real wealth, with 4/5 of a million. The most straight-forward interpretation of (1) the fact that there is within-pair differences in volatility in wealth, and (2) that this variation is mainly composed of volatility in real wealth, is that within-pair differences in wealth is largely driven by differential developments on the housing market. This also alleviates some concerns about remaining confounding from unobserved non-shared environmental variables, since this type of variation can plausibly be assumed to be exogenous.^9^

## Results

Table 3 presents the empirical test of H1a, the main argument presented in the literature on patrimonial voting (the full regression tables can be found in Appendix B). Citizens are expected to become more likely to support free market policies when they grow wealthy since low taxes and less regulations will increase their wealth further. As we can see in columns 1–3, the naive tests support this argument in that the estimate is positive and significant. Even holding observable characteristics constant, more wealthy citizens seem to prefer less regulation. However, this result disappears in columns 4–6 where we add fixed effects at the twin pair level. The coefficients in the discordant models are reduced by roughly 2/3 and are not statistically significant. The reduction when moving from naive to within-models is also itself significant. This strongly indicates that the naive results are driven by unobserved confounders shared by twins, and that a rigorous set of controls capture little or none of this covariation.^10^

**Table 3:** Free market preferences

Free market preferences						
Variables	(1)	(2)	(3)	(4)	(5)	(6)
	Naive	Naive	Naive	Within	Within	Within
Wealth, MKr	0.0228***	0.0259***	0.0276***	0.00877	0.0104	0.00906
	(0.00424)	(0.00455)	(0.00453)	(0.0127)	(0.0137)	(0.0158)
$\varDelta$ Wealth						0.00188
						(0.0132)
Observations	2222	2173	2071	2173	2071	2071
R-squared	0.014	0.031	0.062	0.778	0.786	0.786
Controls	No	Yes	Yes	Yes	Yes	Yes
Occupation FE	No	No	Yes	No	Yes	Yes

Robust standard errors in parentheses

*** p < 0.01, ** p < 0.05, * p < 0.1

**Table 4:** Property tax support

Property tax support						
Variables	(1)	(2)	(3)	(4)	(5)	(6)
	Naive	Naive	Naive	Within	Within	Within
Real wealth, MKr	− 0.102***	− 0.107***	− 0.108***	− 0.0617**	− 0.0682**	− 0.0596*
	(0.00965)	(0.0104)	(0.0108)	(0.0306)	(0.0330)	(0.0339)
$\varDelta$ Wealth						− 0.0111
						(0.00751)
Observations	2299	2249	2143	2249	2143	2143
R-squared	0.050	0.052	0.069	0.717	0.736	0.736
Controls	No	Yes	Yes	Yes	Yes	Yes
Occupation FE	No	No	Yes	No	Yes	Yes

Robust standard errors in parentheses

***p < 0.01, **p < 0.05, *p < 0.1

In contrast, Table 4 presents our most likely case, H1b: the effect of real holdings on preferences for property taxation. Again, the coefficients are sizable and significant and barely budge when introducing the controls. In this case the introduction of twin pair fixed effects also reduces the effect size somewhat, but it does remain statistically significant. Unlike H1a, it thus seems that H1b survives this much more stringent test. The magnitude of the effect is such that one population standard deviation increase in real assets can be expected to lead to a three percentage point reduction in support for real estate taxes (or just above a tenth of a scale step), whereas going from the bottom to the top of the wealth distribution corresponds to just under a whole scale step.

The insurance argument of H2 is tested in Table 5. If this holds up, citizens with large holdings of financial assets should be less supportive of redistribution and social spending given that their large holdings of liquid wealth can be used as an insurance against income losses. Columns 1–3 presents results from naive regressions with and without controls. The only control that makes a dent in the naive estimate is occupational category, but the coefficient is still significant. Within twin-pair models, however, reduce the effect sizes substantially, and now they also fail to reach any level of statistical significance. Here we are again forced to conclude that the naive results connecting financial wealth to redistributive political preferences were largely driven by unobserved familial characteristics.

**Table 5:** Redistributive preferences

Redistributive preferences						
Variables	(1)	(2)	(3)	(4)	(5)	(6)
	Naive	Naive	Naive	Within	Within	Within
Share financial wealth	− 0.106***	− 0.0936***	− 0.0844***	− 0.0398	− 0.0365	− 0.0271
	(0.0147)	(0.0154)	(0.0161)	(0.0413)	(0.0463)	(0.0466)
$\varDelta$ wealth						− 0.00955
						(0.0114)
Observations	2205	2159	2057	2159	2057	2057
R-squared	0.026	0.037	0.063	0.776	0.786	0.787
Controls	No	Yes	Yes	Yes	Yes	Yes
Occupation FE	No	No	Yes	No	Yes	Yes

Robust standard errors in parentheses

***p < 0.01, **p < 0.05, *p < 0.1

Tables 6 and 7 and contain the results for H3. Our expectations are that citizens with more wealth should be more likely to place themselves to the right on the left–right scale and more likely to express support for a right-wing party. This is clearly descriptively true as evidenced by column 1 in both tables, and the coefficients are furthermore only slightly attenuated by the inclusion of the controls. They continue to be attenuated to about 1/2 of the naive effect size when moving to within twin-pair comparisons, and only remain significant at the weakest level reported (10%). The magnitude of the effect is such that going from the bottom to the top of the wealth distribution will push an individual on average less than half a scale step (out of ten) to the right.

So far we’ve seen that the effect sizes change substantially when we use discordant twin models to control for unobservable genetic and common environmental factors. In several cases, the estimates are no longer statistically significant. It is important to note that this loss of significance is also partially driven by the decrease in statistical precision that comes with utilizing within-pair variation rather than cross-pair variation. However, changes that are purely an artefact of reduced precision would be as likely to end up positive as negative. The large reductions of the coefficients, in every case, therefore speaks strongly in favor of there being substantial amounts of unmeasured confounding that is picked up by the discordant MZ twin design, but that largely is left unchecked when using conventional statistical controls.

**Table 6:** Left–right orientation

Left–right orientation						
Variables	(1)	(2)	(3)	(4)	(5)	(6)
	Naive	Naive	Naive	Within	Within	Within
Wealth, MKr	0.0811***	0.0764***	0.0740***	0.0362**	0.0319*	0.0424**
	(0.00578)	(0.00633)	(0.00652)	(0.0180)	(0.0183)	(0.0206)
$\varDelta$ Wealth						− 0.0148
						(0.0154)
Observations	2303	2254	2151	2254	2151	2151
R-squared	0.074	0.079	0.111	0.791	0.805	0.806
Controls	No	Yes	Yes	Yes	Yes	Yes
Occupation FE	No	No	Yes	No	Yes	Yes

Robust standard errors in parentheses

***p < 0.01, **p < 0.05, *p < 0.1

**Table 7:** Party choice (L-R)

Party choice (L–R)						
Variables	(1)	(2)	(3)	(4)	(5)	(6)
	Naive	Naive	Naive	Within	Within	Within
Wealth, MKr	0.107***	0.0934***	0.0913***	0.0476**	0.0442*	0.0439*
	(0.00735)	(0.00798)	(0.00848)	(0.0219)	(0.0226)	(0.0234)
$\varDelta$ Wealth						0.000543
						(0.0185)
Observations	2101	2054	1964	2054	1964	1964
R-squared	0.089	0.094	0.138	0.822	0.838	0.838
Controls	No	Yes	Yes	Yes	Yes	Yes
Occupation FE	No	No	Yes	No	Yes	Yes

Robust standard errors in parentheses

***p < 0.01, **p < 0.05, *p < 0.1

The differences in coefficients between the naive and the within pair estimates is, by definition, attributable to unmeasured confounders of either a genetic or a shared environmental nature. To more specifically investigate the source of this unmeasured confounding, we turn to bivariate ACE modeling and include the sample of dizygotic twins. This allows us to disentangle the sources of the reduction in the effect sizes. The results are presented in Table 8.

**Table 8:** Bivariate ACE decompositions

	$h^{2}$	$c^{2}$	$e^{2}$	biv $h^{2}$	biv $c^{2}$	biv $e^{2}$
Wealth	0.31	0.43	0.27			
	[0.26; 0.35]	[0.38; 0.46]	[0.26; 0.28]			
Free market	0.27	0.15	0.58	0.04	0.06	0.01
	[0.11; 0.43]	[0.01; 0.28]	[0.54; 0.63]	[− 0.02; 0.1]	[0; 0.11]	[0; 0.03]
Left–right	0.16	0.31	0.53	0.02	0.2	0.02
	[0.03; 0.3]	[0.2; 0.41]	[0.49; 0.56]	[− 0.04; 0.07]	[0.17; 0.26]	[0.01; 0.03]
Party	0.18	0.31	0.51	0.06	0.17	0.02
	[0.03; 0.33]	[0.18; 0.43]	[0.47; 0.55]	[0; 0.11]	[0.11; 0.22]	[0.01; 0.03]
Financial wealth	0.43	0.22	0.35			
	[0.37; 0.48]	[0.17; 0.27]	[0.34; 0.37]			
Redistribution	0.27	0.13	0.6	0.01	− 0.12	− 0.02
	[0.1; 0.44]	[− 0.02; 0.27]	[0.56; 0.65]	[− 0.06; 0.08]	[− 0.18; − 0.06]	[− 0.04; − 0.01]
Real wealth	0.26	0.4	0.34			
	[0.21; 0.31]	[0.36; 0.45]	[0.33; 0.35]			
Property taxes	0.22	0.03	0.74	− 0.06	− 0.1	− 0.04
	[0.07; 0.35]	[− 0.02; 0.18]	[0.69; 0.79]	[− 0.12; 0.01]	[− 0.16; − 0.04]	[− 0.05; − 0.02]

Each vertical segment in the table represents one wealth type, and its accompanying political preference variables with bivariate decompositions

The first thing to note are the univariate estimates of the share of variation that can be attributed to additive genetics (heritability, or $h^{2}$), shared environment ($c^{2}$) and unique environment ($e^{2}$). Wealth comes in at a heritability of 31% (which is in line with Benjamin et al. (2012)), subdivided into 43% for financial wealth and 26% for real wealth. Of the political outcomes, we see a heritability of 27% for redistributive and free-market preferences, with most of the remainder being picked up by unique environmental influences and very little by shared environment (13 and 15% respectively, with the former not being statistically distinguishable from zero). In contrast, the more “identity” oriented measures (left–right placement and party preference) show heritabilities of a mere 16 and 18 %, with a substantially larger share (31%) picked up by shared environment. For all political outcomes, unique environment picks up a majority of the variation. It is important to keep in mind that the unique environmental term represents all influences that serve to make a pair of twins *less* similar to each other. This includes the influence from all life experiences that they do not share, but importantly also captures all measurement error. Considering that measures of political attitudes such as the ones employed here will be somewhat noisily measured—in particular left–right placement and party choice since they are based on single survey questions—it is likely that both $h^{2}$ and $c^{2}$ are slightly deflated.

Moving to the main point, the sources of the unmeasured confounding picked up by shared twin factors can be seen in the columns named biv $h^{2}$, biv $c^{2}$ and biv $e^{2}$. For all wealth and outcome combinations, the bivariate shared environment component (biv $c^{2}$) picks up the most covariance (almost always by a wide margin). This factor appears particularly strong for left–right placement and party choice in comparison to free market preferences, redistributive preferences and property tax preferences. Such a difference could indicate that the shared environmental factors that influence wealth acquisition are also more strongly related to political identity (which is more clearly tapped by left–right placement and party choice) than to specific political issue attitudes. In no cases are the bivariate heritabilities statistically distinguishable from zero, indicating that genetic confounding may not be as big of an issue as unmeasured environmental confounders shared within families.

## Discussion

A popular argument in political science is that the wealthy practice patrimonial voting in that they vote for right-wing parties to increase their fortunes. While this pattern is well documented, the underlying mechanism (if causal or not) is not fully understood. Descriptively speaking, wealthier people in this sample of Swedish middle-aged twin pairs are more supportive of free market policies, less supportive of redistribution and real-estate taxes, and more likely to identify as right-wing and vote for right-wing parties. Causally speaking, however, the effect of wealth (all or financial) on domain specific policy preferences about taxes and market regulations on the one hand, or questions about economic redistribution on the other, appear to be much more modest than ordinarily assumed, and are not statistically significant.

When moving to within-twin pair analyses it is revealed that naive correlations, rather than reflecting a true causal signal, are likely severely biased by unobservable familial characteristics. This bias is largely left unchecked by conventional statistical controls such as income, education, family size or occupational category, which underscores the importance of taking familial confounders into account. Bivariate variance decomposition models further showed that the sources of this confounding are more likely shared family environment than genetic overlap.

While previous studies using lottery-winnings have shown some effects on political preferences, it should be noted that these studies concern a fairly dramatic relative increase in net wealth. Most voters are unlikely to experience a wealth shock comparable to the magnitude of a large lottery prize sum, not even under extraordinary circumstances such as financial crises or recessions. If our results are quantitatively transferable, we should expect few individuals to significantly alter their views on most economic and social policy issues in response to a realistic change in individual net wealth. However—we *do* find remaining effects for a few outcomes even after taking familial confounders into account, although these findings are mostly weak in both a substantive and statistical sense. Most notably, the highly specific issue of property taxation appears to be an exception to the pattern above.

This finding falls in line with studies that find a general link between property ownership and resistance against property taxation (Brunner et al. 2015). As argued in the theory section, we think that the case of property taxes and real wealth, in this particular setting, should be seen as a type of most likely case for finding an effect. People can fail to precisely compute the general costs and benefits of government policy (as would often be required for the free-market and redistribution dimensions), and yet find this specific form of taxation of their property obtrusive. The connection between the value of ones’ house rising, and paying larger amounts in property taxes, is hard to miss.

We do find some remaining effects, although statistically weak, on self-reported left–right placement and party choice. These effect sizes are similarly highly attenuated when controlling for familial confounders. It is possible that these two indicators are picking up the effect from other pocketbook type mechanisms than those captured by the survey items included in our issue dimensions. For instance, the right-wing coalition abolished the wealth tax at the time of the survey and Swedish property owners were saved from collapsing house prices as the conservative administration was able to mitigate much of the impact of the financial crisis. Unfortunately, no survey items regarding these policy preferences were available.

There are, as always, a few concerns about the interpretation of the lack of results when using a discordant twin design. First, if twins tend to exert a marked influence on each other (as adults) in a way that is relevant to the variables of interest, this will violate independence assumptions and introduce bias. The bias can go in either direction depending on whether this effect makes the twins more or less similar (i.e. if they come to diverge into different behavioral niches as a consequence). One particular way in which this could function in the present case is if the type of self-interest mechanisms we’re interested in testing extends within the family—specifically to the other twin. If so, this may dampen within-pair effects if one twin adapts, for example, to prefer less taxation as his or her cotwin gets richer. Robustness checks using self-reported contact rates between the twins in Appendix A give no indication that this is a problem for our results, but these tests do not completely rule out this potential design weakness.

Another remaining possible limitation is concerns about reverse causality or collider bias: values might affect wealth acquisition, and may also affect some of the controls. Collider effects would bias the estimates downward, but do not appear to be an issue since the introduction of controls has only minor consequences for the estimates. Reverse causality on the other hand would not lead to any of the estimates being biased up or down, but would obviously render moot any causal conclusions for the results that remain. In the absence of fine-grained longitudinal data for political attitudes, we will have to rely on plausibility: it appears dramatically less likely, for example, that having a particular opinion about real-estate taxes causes the value of one’s house to change, rather than the other way around.

Something should be said about the implications of the bivariate ACE models for future observational research. As mentioned in the methods section, these results indicate, at least in the current setting, where to look for confounding factors to include in the models. The absence of any substantial genetic confounding in these relationships indicate that we can conceivably get by without genetically informed data—provided that the sources of the environmental confounding can be identified and controlled for. The largest part of this confounding appears to stem from shared environmental factors—possibly family environment or shared networks. If these are accurately captured, observational models may be able to avoid a large amount of the endogeneity problem that we document here.

Finally, it is difficult to conceive of reasons why the external validity of these findings should be confined specifically to twins, since it would require that having a twin in itself nullifies most of the causal relationship between wealth and preferences over government policy. The results are therefore likely generalizable to a wider Swedish context, at the very least for the cohorts included in the study. Another question is how far these results can travel in an international context given that the universal welfare system in Sweden reduces the need for private savings in times of financial distress. However, recent research suggests that this context likely constitutes a tough test for the patrimonial voting hypothesis in that a larger effect of asset wealth on voting is found in more liberal welfare systems like the UK (Quinlan and Okolikj 2019). Thus, we think it is reasonable to assume that our results are generalizable to most other OECD-nations.

Our results should encourage scholars to investigate the relationship between economic resources and political preferences further. We find, as others have before us, that there is a descriptive link between wealth and political attitudes and behavior. However, it seems that these naive comparisons—even when using a rich set of statistical controls—overestimate the causal effect of wealth quite substantially, if there is one at all. We suggest that future research should aim at finding situations where the economic stakes are clear and unobtrusive to voters, and where there is an unambiguous connection between the policy issue at hand and the immediate self-interest of asset holders. Further research may provide important new knowledge about the effects of increasing wealth inequality, as well as insights into processes of political preference formation.

## Supplementary information

Below is the link to the electronic supplementary material.

Supplementary material 1 (pdf 161 KB)

## Footnotes

1 We also test this hypothesis specifically with financial wealth. These results are reported in Appendix C, but are consistent with our main test.

2 The R package *umx* (Bates et al. 2019), a wrapper for *OpenMx*, was used for all decomposition models.

3 Replication materials can be found at 10.7910/DVN/JMUWNE

4 Free schools are privately run (and often for profit) schools included in a public voucher system.

5 The parties are traditionally ordered in the following way, from left to right: Left party, Social democracts, Greens, Center party, Liberal party, Christian Democrats, Moderates, Sweden Democrats.

6 Additional results for outcomes constructed from the first five principal components in the preference data are reported in Appendix D.

7 Trimming was used rather than Winsorising since the data is from registries, and outliers will therefore not be due to misreporting.

8 Appendix E also contains a comparison between the MZ twin sample and the general population across the same cohorts, for a few variables that we had access to. The twin sample has a slightly higher level of education (the equivalent of about 5 months extra schooling), and higher income (16% higher). There is also a higher proportion of women in the twin sample. While there may be reasons to think that wealth effects are heterogeneous over things like income, the differences in this regard should not be large enough to pose serious problems for the external validity of the results. Furthermore, the political preferences of the twins, as evidenced by a comparison between their self-reported party choice and the equivalent election results, appear to be well in line with those of the general population.

9 An anonymous reviewer suggested that some of the difference in wealth trajectories within pairs might be driven by one of the twins marrying into wealth. Due to the age of the sample (the participants in the SALTY survey were 51–66 years of age when their attitudes were measured, and consequently 41–56 at the start of the wealth measurement period) this is not likely to be the case. Our data suggests that no more than 90 individuals in the effective MZ sample got married during this period.

10 The careful reader will notice the large increase in R-squared in the within models. This is an artefact of using within-pair variation: since the pair fixed effects will soak up all of the variation caused by factors that the twins share (i.e. both genetic effects and shared environment), the R-squared is bound to increase substantially when moving from naive to within-pair models.
