# Supplementary material for: Uncovering the Source of Patrimonial Voting: Evidence from Swedish Twin Pairs
Source: Polit Behav. 2021 Jan 30;44(4):1681–702. doi: 10.1007/s11109-020-09669-4 (PMC9674710; doi:10.1007/s11109-020-09669-4)
Supplement: Supplementary file 1 — Supplementary material 1 (pdf 161 KB) [file 11109_2020_9669_MOESM1_ESM.pdf]

Online appendix for: Uncovering the Source of Patrimonial Voting:

Evidence from Swedish Twin Pairs

Rafael Ahlskog and Anton Brännlund

Appendix A: robustness checks

Inverse hyperbolic sine of wealth

As a robustness check to see to what extent the skew in the distribution of wealth affects the results, we here report the same models with the hyperbolic sine of wealth rather than raw wealth. Results from these models are broadly consistent with the main results.

Table A.1:
Free market preferences

VARIABLES	(1) Naive	(2) Naive	(3) Naive	(4) Within	(5) Within	(6) Within
H/S wealth	0.0363***	0.0427***	0.0460***	0.0171	0.0207	0.0205
	(0.00705)	(0.00750)	(0.00755)	(0.0202)	(0.0220)	(0.0257)
Δ wealth						0.000230
						(0.0132)
Observations	2,222	2,173	2,071	2,173	2,071	2,071
R-squared	0.013	0.031	0.063	0.778	0.787	0.787
Controls	No	Yes	Yes	Yes	Yes	Yes
Occupation FE	No	No	Yes	No	Yes	Yes

Robust standard errors in parentheses
*** p<0.01, ** p<0.05, * p<0.1

Table A.2:
Property tax support

VARIABLES	(1) Naive	(2) Naive	(3) Naive	(4) Within	(5) Within	(6) Within
H/S real wealth	-0.153***	-0.162***	-0.163***	-0.103**	-0.113**	-0.102**
	(0.0136)	(0.0147)	(0.0152)	(0.0414)	(0.0450)	(0.0461)
Δ wealth						-0.00996
						(0.00717)
Observations	2,299	2,249	2,143	2,249	2,143	2,143
R-squared	0.056	0.058	0.076	0.719	0.737	0.738
Controls	No	Yes	Yes	Yes	Yes	Yes
Occupation FE	No	No	Yes	No	Yes	Yes

Robust standard errors in parentheses
*** p<0.01, ** p<0.05, * p<0.1

Table A.3:
Redistributive preferences

VARIABLES	(1) Naive	(2) Naive	(3) Naive	(4) Within	(5) Within	(6) Within
H/S financial wealth	-0.123***	-0.109***	-0.0987***	-0.0514	-0.0482	-0.0374
	(0.0163)	(0.0170)	(0.0178)	(0.0475)	(0.0532)	(0.0536)
Δ wealth						-0.00928
						(0.0114)
Observations	2,205	2,159	2,057	2,159	2,057	2,057
R-squared	0.028	0.038	0.064	0.776	0.786	0.787
Controls	No	Yes	Yes	Yes	Yes	Yes
Occupation FE	No	No	Yes	No	Yes	Yes

Robust standard errors in parentheses
*** p<0.01, ** p<0.05, * p<0.1

Table A.4:
Party choice (L-R)

VARIABLES	(1) Naive	(2) Naive	(3) Naive	(4) Within	(5) Within	(6) Within
H/S wealth	0.179***	0.157***	0.154***	0.0699*	0.0701*	0.0692*
	(0.0124)	(0.0134)	(0.0141)	(0.0362)	(0.0375)	(0.0393)
Δ wealth						0.000863
						(0.0185)
Observations	2,101	2,054	1,964	2,054	1,964	1,964
R-squared	0.093	0.097	0.141	0.822	0.838	0.838
Controls	No	Yes	Yes	Yes	Yes	Yes
Occupation FE	No	No	Yes	No	Yes	Yes

Robust standard errors in parentheses
*** p<0.01, ** p<0.05, * p<0.1

Table A.5:
Left-right orientation

VARIABLES	(1) Naive	(2) Naive	(3) Naive	(4) Within	(5) Within	(6) Within
H/S wealth	0.134***	0.125***	0.122***	0.0588**	0.0540*	0.0715**
	(0.00985)	(0.0107)	(0.0110)	(0.0297)	(0.0307)	(0.0347)
Δ wealth						-0.0158
						(0.0153)
Observations	2,303	2,254	2,151	2,254	2,151	2,151
R-squared	0.074	0.079	0.112	0.792	0.806	0.806
Controls	No	Yes	Yes	Yes	Yes	Yes
Occupation FE	No	No	Yes	No	Yes	Yes

Robust standard errors in parentheses
*** p<0.01, ** p<0.05, * p<0.1

Contact rate interactions

To address concerns that depression of effect sizes in within-pair models are due to expectations on the part of one twin to take part in the wealth of the other twin and updating preferences on that basis, we report within-pair models where wealth is interacted with the self-reported contact rate between the twins (measured as number of times per year the two are in contact). More specifically, this is accomplished by differencing all variables in the model within pairs, generating difference scores, and interacting these difference scores for the independent variable and all the controls with the contact rate. Note that this generates observations at the pair level rather than individual level - the lower number of observations reported are thus due to each observation representing a pair. The controls are the standard set, without occupation, making the models equivalent to those reported in the fourth column of the main results.

The expectation is that if the above mentioned dynamic does in fact bias our results, a) the coefficients conditional on zero contact should be further away from zero than when this interaction is not included, and b) the interaction coefficients should be significant (table A.6). The results show that the sizes of the wealth coefficients conditional on zero contact are broadly consistent with the uninteracted effect sizes reported in the main text – the exception being for left-right placement, which is *closer* to zero rather than further away from zero. Furthermore, none of the interactions are significant.

Table A.6:

VARIABLES	(1) Free market	(2) Property tax	(3) Redistribution	(4) Left-right	(5) Party
Contact rate	4.45e-05	-0.000129	-8.49e-05*	6.41e-05	0.000237***
	(4.84e-05)	(9.66e-05)	(4.96e-05)	(6.98e-05)	(8.93e-05)
Wealth	0.00554			0.0211	0.0516**
	(0.0114)			(0.0165)	(0.0201)
Wealth × Contact	8.19e-06			9.57e-05	-6.43e-05
	(5.90e-05)			(8.70e-05)	(0.000105)
Real wealth		-0.0601**			
		(0.0269)			
Real wealth × Contact		-7.61e-07			
		(0.000136)			
Financial wealth			-0.0821**		
			(0.0403)		
Financial wealth × Contact			0.000258		
			(0.000206)		
Observations	827	872	813	878	742
R-squared	0.017	0.021	0.027	0.029	0.032
Controls	Yes	Yes	Yes	Yes	Yes

Standard errors in parentheses
*** p<0.01, ** p<0.05, * p<0.1

Appendix B: full models, main hypotheses

Table B.1:
Free market preferences

VARIABLES	(1) Naive	(2) Naive	(3) Naive	(4) Within	(5) Within	(6) Within
Wealth, MKr	0.0228***	0.0259***	0.0276***	0.00877	0.0104	0.00906
	(0.00424)	(0.00455)	(0.00453)	(0.0127)	(0.0137)	(0.0158)
Sex of twin		0.0401***	0.0346***			
		(0.00898)	(0.0106)			
Work income		0.0345	0.0532*	0.0225	0.0289	0.0277
		(0.0257)	(0.0291)	(0.0657)	(0.0759)	(0.0765)
Education years		-0.00751***	-0.00661***	-0.00311	-0.00319	-0.00318
		(0.00176)	(0.00222)	(0.00577)	(0.00660)	(0.00661)
Children under 18 = 1		-0.0175	-0.0173	0.0127	0.0185	0.0184
		(0.0183)	(0.0193)	(0.0395)	(0.0444)	(0.0444)
Children under 18 = 2		-0.0108	-0.0180	-0.0406	-0.0387	-0.0394
		(0.0299)	(0.0299)	(0.0395)	(0.0417)	(0.0420)
Children under 18 = 3		0.0185	0.0325	0.353***	0.388***	0.386***
		(0.103)	(0.108)	(0.0586)	(0.0845)	(0.0851)
Occupation = 11			-0.0849		-0.00755	-0.00850
			(0.129)		(0.171)	(0.170)
Occupation = 12			-0.0651		0.00856	0.00854
			(0.0405)		(0.0911)	(0.0910)
Occupation = 13			-0.0578		0.0311	0.0308
			(0.0448)		(0.102)	(0.102)
Occupation = 21			-0.122***		-0.0331	-0.0333
			(0.0424)		(0.0974)	(0.0974)
Occupation = 22			-0.0524		0.0132	0.0129
			(0.0455)		(0.102)	(0.102)
Occupation = 23			-0.0855**		0.00691	0.00676
			(0.0416)		(0.0930)	(0.0929)
Occupation = 24			-0.117***		-0.0116	-0.0116
			(0.0410)		(0.0906)	(0.0905)
Occupation = 31			-0.119***		-0.00281	-0.00288
			(0.0411)		(0.0951)	(0.0950)
Occupation = 32			-0.0911**		0.0172	0.0168
			(0.0432)		(0.0972)	(0.0972)
Occupation = 33			-0.122***		-0.00859	-0.00878
			(0.0434)		(0.110)	(0.110)
Occupation = 34			-0.0899**		-0.0187	-0.0189
			(0.0398)		(0.0868)	(0.0868)
Occupation = 41			-0.0703*		-0.0228	-0.0231
			(0.0403)		(0.0904)	(0.0904)
Occupation = 42			0.0231		0.0403	0.0404
			(0.0469)		(0.110)	(0.110)
Occupation = 51			-0.0768*		0.00662	0.00663
			(0.0395)		(0.0903)	(0.0902)
Occupation = 52			-0.0418		0.0418	0.0417
			(0.0456)		(0.107)	(0.107)
Occupation = 61			-0.0334		0.0769	0.0754
			(0.0681)		(0.120)	(0.122)
Occupation = 71			-0.0967**		-0.0318	-0.0316
			(0.0435)		(0.0981)	(0.0982)
Occupation = 72			-0.136***		-0.0700	-0.0698
			(0.0455)		(0.100)	(0.100)
Occupation = 73			-0.0749		0.00681	0.00656
			(0.0513)		(0.120)	(0.120)
Occupation = 74			0.0645		0.0999	0.100
			(0.0813)		(0.130)	(0.130)
Occupation = 81			-0.158***		-0.0596	-0.0605
			(0.0505)		(0.128)	(0.128)
Occupation = 82			-0.122***		0.00563	0.00530
			(0.0440)		(0.101)	(0.101)
Occupation = 83			-0.0840*		-0.0300	-0.0299
			(0.0472)		(0.118)	(0.118)
Occupation = 91			-0.0814*		-0.0371	-0.0369
			(0.0430)		(0.0976)	(0.0975)
Occupation = 93			-0.139**		-0.0632	-0.0641
			(0.0566)		(0.153)	(0.153)
Sex of twin = o,				-	-	-
Δ wealth						0.00188
						(0.0132)
Constant	0.449***	0.500***	0.569***	0.490***	0.491***	0.492***
	(0.00601)	(0.0207)	(0.0501)	(0.0687)	(0.123)	(0.123)
Observations	2,222	2,173	2,071	2,173	2,071	2,071
R-squared	0.014	0.031	0.062	0.778	0.786	0.786

Robust standard errors in parentheses
*** p<0.01, ** p<0.05, * p<0.1

Table B.2:
Property tax support

VARIABLES	(1) Naive	(2) Naive	(3) Naive	(4) Within	(5) Within	(6) Within
Real wealth, MKr	-0.102***	-0.107***	-0.108***	-0.0617**	-0.0682**	-0.0596*
	(0.00965)	(0.0104)	(0.0108)	(0.0306)	(0.0330)	(0.0339)
Sex of twin		0.00348	0.0136			
		(0.0152)	(0.0190)			
Work income		0.00757	0.0144	-0.00561	0.0216	0.0362
		(0.0430)	(0.0494)	(0.129)	(0.146)	(0.147)
Education years		0.00484*	0.00875**	0.0142	0.0172	0.0168
		(0.00286)	(0.00376)	(0.00997)	(0.0123)	(0.0123)
Children under 18 = 1		-0.0202	-0.0112	-0.0174	-0.0196	-0.0118
		(0.0320)	(0.0328)	(0.0688)	(0.0767)	(0.0775)
Children under 18 = 2		-0.0514	-0.0668	-0.00343	-0.00517	-0.00191
		(0.0447)	(0.0432)	(0.124)	(0.122)	(0.122)
Children under 18 = 3		0.141	0.144	0.0244	-0.00658	-0.00139
		(0.153)	(0.143)	(0.0548)	(0.130)	(0.130)
Occupation = 11			0.394**		0.176	0.175
			(0.194)		(0.426)	(0.427)
Occupation = 12			0.187*		0.154	0.152
			(0.0980)		(0.219)	(0.221)
Occupation = 13			0.111		-0.0778	-0.0999
			(0.102)		(0.246)	(0.247)
Occupation = 21			0.168*		0.0845	0.0844
			(0.1000)		(0.223)	(0.225)
Occupation = 22			0.0875		-0.00834	-0.00633
			(0.100)		(0.233)	(0.235)
Occupation = 23			0.197**		0.0823	0.0819
			(0.0973)		(0.220)	(0.222)
Occupation = 24			0.156		0.0260	0.0235
			(0.0969)		(0.218)	(0.220)
Occupation = 31			0.195**		0.228	0.230
			(0.0988)		(0.223)	(0.225)
Occupation = 32			0.132		0.00129	0.00192
			(0.0988)		(0.225)	(0.226)
Occupation = 33			0.177*		0.126	0.128
			(0.101)		(0.229)	(0.231)
Occupation = 34			0.159*		0.0533	0.0530
			(0.0954)		(0.216)	(0.218)
Occupation = 41			0.166*		0.0660	0.0666
			(0.0964)		(0.219)	(0.221)
Occupation = 42			0.235**		0.0996	0.0998
			(0.110)		(0.248)	(0.250)
Occupation = 51			0.184*		0.0634	0.0629
			(0.0960)		(0.217)	(0.219)
Occupation = 52			0.175*		0.103	0.116
			(0.105)		(0.234)	(0.237)
Occupation = 61			0.252*		0.200	0.204
			(0.133)		(0.354)	(0.353)
Occupation = 71			0.267***		0.170	0.169
			(0.0994)		(0.227)	(0.229)
Occupation = 72			0.175*		0.0703	0.0683
			(0.103)		(0.242)	(0.244)
Occupation = 73			0.269**		-0.0171	-0.0151
			(0.134)		(0.299)	(0.299)
Occupation = 74			-0.131		0.0559	0.0537
			(0.133)		(0.438)	(0.443)
Occupation = 81			0.190		0.0385	0.0388
			(0.121)		(0.338)	(0.338)
Occupation = 82			0.239**		0.110	0.111
			(0.102)		(0.237)	(0.238)
Occupation = 83			0.218**		0.0529	0.0523
			(0.104)		(0.272)	(0.274)
Occupation = 91			0.187*		0.0674	0.0652
			(0.101)		(0.236)	(0.237)
Occupation = 93			0.162		-0.00305	-0.00150
			(0.133)		(0.310)	(0.308)
Sex of twin = o,				-	-	-
Δ wealth						-0.0111
						(0.00751)
Constant	0.468***	0.410***	0.177	0.272**	0.154	0.155
	(0.00977)	(0.0340)	(0.109)	(0.124)	(0.274)	(0.276)
Observations	2,299	2,249	2,143	2,249	2,143	2,143
R-squared	0.050	0.052	0.069	0.717	0.736	0.736

Robust standard errors in parentheses
*** p<0.01, ** p<0.05, * p<0.1

Table B.3:
Redistributive preferences

VARIABLES	(1) Naive	(2) Naive	(3) Naive	(4) Within	(5) Within	(6) Within
Financial wealth, MKr	-0.106***	-0.0936***	-0.0844***	-0.0398	-0.0365	-0.0271
	(0.0147)	(0.0154)	(0.0161)	(0.0413)	(0.0463)	(0.0466)
Sex of twin		0.0126	0.00794			
		(0.00903)	(0.0112)			
Work income		-0.0801***	-0.0801**	-0.0338	-0.0267	-0.0242
		(0.0270)	(0.0315)	(0.0682)	(0.0787)	(0.0794)
Education years		-0.000664	-0.00210	-0.00534	-0.00627	-0.00611
		(0.00173)	(0.00219)	(0.00586)	(0.00655)	(0.00662)
Children under 18 = 1		0.0395*	0.0417*	0.0264	0.0144	0.0174
		(0.0210)	(0.0217)	(0.0376)	(0.0397)	(0.0395)
Children under 18 = 2		0.00842	0.0220	0.0650	0.0715	0.0759
		(0.0315)	(0.0310)	(0.0488)	(0.0513)	(0.0520)
Children under 18 = 3		0.00316	-0.0176	-0.250***	-0.266***	-0.263***
		(0.0842)	(0.0844)	(0.0445)	(0.0626)	(0.0633)
Occupation = 11			0.245		0.252	0.258
			(0.163)		(0.184)	(0.184)
Occupation = 12			0.0946*		0.0116	0.0162
			(0.0548)		(0.125)	(0.125)
Occupation = 13			0.0834		-0.0326	-0.0202
			(0.0579)		(0.135)	(0.137)
Occupation = 21			0.115**		0.0476	0.0509
			(0.0563)		(0.132)	(0.133)
Occupation = 22			0.117**		0.0606	0.0622
			(0.0573)		(0.129)	(0.129)
Occupation = 23			0.129**		0.0549	0.0588
			(0.0549)		(0.129)	(0.129)
Occupation = 24			0.134**		0.0378	0.0405
			(0.0549)		(0.125)	(0.126)
Occupation = 31			0.117**		0.0674	0.0727
			(0.0542)		(0.129)	(0.130)
Occupation = 32			0.160***		0.0853	0.0875
			(0.0555)		(0.128)	(0.128)
Occupation = 33			0.163***		0.0797	0.0836
			(0.0562)		(0.135)	(0.135)
Occupation = 34			0.107**		0.0567	0.0615
			(0.0533)		(0.121)	(0.122)
Occupation = 41			0.0989*		0.0571	0.0627
			(0.0539)		(0.124)	(0.125)
Occupation = 42			0.0495		0.0890	0.0902
			(0.0581)		(0.142)	(0.142)
Occupation = 51			0.143***		0.0524	0.0560
			(0.0534)		(0.125)	(0.125)
Occupation = 52			0.0915		0.0320	0.0354
			(0.0596)		(0.137)	(0.137)
Occupation = 61			0.0570		-0.0690	-0.0442
			(0.0678)		(0.170)	(0.169)
Occupation = 71			0.132**		0.0464	0.0493
			(0.0562)		(0.129)	(0.130)
Occupation = 72			0.171***		0.0801	0.0842
			(0.0562)		(0.141)	(0.142)
Occupation = 73			0.101		-0.0384	-0.0328
			(0.0654)		(0.189)	(0.190)
Occupation = 74			0.0615		-0.0415	-0.0465
			(0.0965)		(0.143)	(0.146)
Occupation = 81			0.174***		0.0569	0.0646
			(0.0665)		(0.153)	(0.153)
Occupation = 82			0.144**		0.0330	0.0389
			(0.0567)		(0.130)	(0.130)
Occupation = 83			0.145**		0.0439	0.0485
			(0.0583)		(0.141)	(0.142)
Occupation = 91			0.123**		0.0543	0.0564
			(0.0568)		(0.130)	(0.131)
Occupation = 93			0.120**		0.00512	0.0143
			(0.0588)		(0.156)	(0.156)
Sex of twin = o,				-	-	-
Δ wealth						-0.00955
						(0.0114)
Constant	0.616***	0.643***	0.540***	0.680***	0.641***	0.639***
	(0.00513)	(0.0205)	(0.0612)	(0.0711)	(0.150)	(0.151)
Observations	2,205	2,159	2,057	2,159	2,057	2,057
R-squared	0.026	0.037	0.063	0.776	0.786	0.787

Robust standard errors in parentheses
*** p<0.01, ** p<0.05, * p<0.1

Table B.4:

Party choice (L-R)

VARIABLES	(1)<br>Naive	(2)<br>Naive	(3)<br>Naive	(4)<br>Within	(5)<br>Within	(6)<br>Within
Wealth, MKr	0.107***	0.0934***	0.0913***	0.0476**	0.0442*	0.0439*
	(0.00735)	(0.00798)	(0.00848)	(0.0219)	(0.0226)	(0.0234)
Sex of twin		0.0150	0.00290			
		(0.0168)	(0.0201)			
Work income		0.142***	0.104**	-0.000554	0.0637	0.0634
		(0.0461)	(0.0527)	(0.116)	(0.133)	(0.134)
Education years		0.00639**	0.00224	-0.00953	-0.00722	-0.00722
		(0.00322)	(0.00409)	(0.00982)	(0.0112)	(0.0112)
Children under 18 = 1		-0.0781**	-0.0754**	-0.0578	-0.0437	-0.0437
		(0.0321)	(0.0329)	(0.0739)	(0.0806)	(0.0806)
Children under 18 = 2		-0.00118	-0.0351	-0.0407	-0.0288	-0.0290
		(0.0530)	(0.0509)	(0.0639)	(0.0789)	(0.0794)
Children under 18 = 3		0.179	0.221*	0.348***	0.395*	0.394*
		(0.116)	(0.116)	(0.0877)	(0.234)	(0.235)
Occupation = 11			-0.565***		-0.325	-0.325
			(0.194)		(0.291)	(0.291)
Occupation = 12			-0.342***		-0.424*	-0.424*
			(0.0456)		(0.245)	(0.245)
Occupation = 13			-0.298***		-0.287	-0.287
			(0.0658)		(0.254)	(0.254)
Occupation = 21			-0.369***		-0.379	-0.379
			(0.0513)		(0.246)	(0.246)
Occupation = 22			-0.313***		-0.492**	-0.492*
			(0.0542)		(0.250)	(0.251)
Occupation = 23			-0.362***		-0.470*	-0.470*
			(0.0463)		(0.244)	(0.244)
Occupation = 24			-0.354***		-0.421*	-0.421*
			(0.0459)		(0.238)	(0.238)
Occupation = 31			-0.390***		-0.396	-0.396
			(0.0500)		(0.244)	(0.244)
Occupation = 32			-0.424***		-0.530**	-0.530**
			(0.0490)		(0.245)	(0.245)
Occupation = 33			-0.420***		-0.453*	-0.453*
			(0.0543)		(0.246)	(0.246)
Occupation = 34			-0.334***		-0.429*	-0.429*
			(0.0447)		(0.236)	(0.236)
Occupation = 41			-0.325***		-0.423*	-0.423*
			(0.0464)		(0.241)	(0.241)
Occupation = 42			-0.214***		-0.430	-0.430
			(0.0632)		(0.265)	(0.265)
Occupation = 51			-0.420***		-0.443*	-0.443*
			(0.0441)		(0.238)	(0.239)
Occupation = 52			-0.324***		-0.334	-0.334
			(0.0606)		(0.257)	(0.257)
Occupation = 61			-0.335***		-0.218	-0.219
			(0.0835)		(0.323)	(0.324)
Occupation = 71			-0.466***		-0.469*	-0.469*
			(0.0508)		(0.242)	(0.242)
Occupation = 72			-0.521***		-0.501**	-0.501**
			(0.0602)		(0.255)	(0.255)
Occupation = 73			-0.212**		-0.312	-0.312
			(0.103)		(0.248)	(0.249)
Occupation = 74			-0.184		-0.433	-0.433
			(0.181)		(0.280)	(0.281)
Occupation = 81			-0.507***		-0.637**	-0.637**
			(0.0867)		(0.320)	(0.320)
Occupation = 82			-0.468***		-0.479*	-0.479*
			(0.0537)		(0.252)	(0.252)
Occupation = 83			-0.466***		-0.478*	-0.478*
			(0.0583)		(0.253)	(0.254)
Occupation = 91			-0.445***		-0.348	-0.348
			(0.0538)		(0.248)	(0.248)
Occupation = 93			-0.558***		-0.654**	-0.655**
			(0.0754)		(0.325)	(0.325)
Sex of twin = o,				-	-	-
Δ wealth						0.000543
						(0.0185)
Constant	0.350***	0.227***	0.680***	0.520***	0.905***	0.905***
	(0.0109)	(0.0365)	(0.0698)	(0.119)	(0.272)	(0.272)
Observations	2,101	2,054	1,964	2,054	1,964	1,964
R-squared	0.089	0.094	0.138	0.822	0.838	0.838

Robust standard errors in parentheses
*** p<0.01, ** p<0.05, * p<0.1

Table B.5:
Left-right orientation

VARIABLES	(1) Naive	(2) Naive	(3) Naive	(4) Within	(5) Within	(6) Within
Wealth, MKr	0.0811***	0.0764***	0.0740***	0.0362**	0.0319*	0.0424**
	(0.00578)	(0.00633)	(0.00652)	(0.0180)	(0.0183)	(0.0206)
Sex of twin		0.0373***	0.0280*			
		(0.0134)	(0.0154)			
Work income		0.137***	0.106**	0.0992	0.0822	0.0921
		(0.0370)	(0.0424)	(0.0879)	(0.0994)	(0.0989)
Education years		-0.00151	-0.00482	-0.00531	-0.00621	-0.00636
		(0.00260)	(0.00335)	(0.00799)	(0.00923)	(0.00917)
Children under 18 = 1		-0.0370	-0.0333	-0.0116	-0.00879	-0.00708
		(0.0251)	(0.0262)	(0.0584)	(0.0615)	(0.0615)
Children under 18 = 2		0.0351	0.0202	0.00677	0.00965	0.0156
		(0.0392)	(0.0401)	(0.0764)	(0.0883)	(0.0886)
Children under 18 = 3		0.106	0.149	0.350***	0.383***	0.392***
		(0.128)	(0.138)	(0.122)	(0.131)	(0.133)
Occupation = 11			-0.511***		-0.389*	-0.381*
			(0.0873)		(0.212)	(0.210)
Occupation = 12			-0.288***		-0.264	-0.264
			(0.0483)		(0.185)	(0.185)
Occupation = 13			-0.234***		-0.161	-0.159
			(0.0570)		(0.200)	(0.202)
Occupation = 21			-0.332***		-0.362*	-0.361*
			(0.0547)		(0.195)	(0.196)
Occupation = 22			-0.261***		-0.367*	-0.364*
			(0.0541)		(0.189)	(0.190)
Occupation = 23			-0.294***		-0.329*	-0.327*
			(0.0495)		(0.190)	(0.191)
Occupation = 24			-0.286***		-0.298	-0.299
			(0.0492)		(0.182)	(0.183)
Occupation = 31			-0.307***		-0.298	-0.297
			(0.0513)		(0.191)	(0.192)
Occupation = 32			-0.335***		-0.344*	-0.342*
			(0.0505)		(0.189)	(0.190)
Occupation = 33			-0.340***		-0.382**	-0.380**
			(0.0555)		(0.191)	(0.192)
Occupation = 34			-0.275***		-0.308*	-0.307*
			(0.0476)		(0.182)	(0.183)
Occupation = 41			-0.263***		-0.307*	-0.307
			(0.0489)		(0.186)	(0.187)
Occupation = 42			-0.238***		-0.329	-0.330
			(0.0578)		(0.215)	(0.216)
Occupation = 51			-0.326***		-0.340*	-0.340*
			(0.0481)		(0.186)	(0.187)
Occupation = 52			-0.284***		-0.325	-0.324
			(0.0617)		(0.206)	(0.207)
Occupation = 61			-0.239***		-0.0794	-0.0678
			(0.0684)		(0.284)	(0.284)
Occupation = 71			-0.366***		-0.364*	-0.366*
			(0.0562)		(0.192)	(0.192)
Occupation = 72			-0.416***		-0.469**	-0.471**
			(0.0582)		(0.212)	(0.213)
Occupation = 73			-0.204**		-0.246	-0.245
			(0.0840)		(0.215)	(0.215)
Occupation = 74			-0.274*		-0.304	-0.308
			(0.157)		(0.213)	(0.213)
Occupation = 81			-0.387***		-0.314	-0.308
			(0.0727)		(0.204)	(0.206)
Occupation = 82			-0.422***		-0.377*	-0.375*
			(0.0527)		(0.198)	(0.198)
Occupation = 83			-0.367***		-0.327	-0.328
			(0.0557)		(0.218)	(0.219)
Occupation = 91			-0.371***		-0.321*	-0.323*
			(0.0548)		(0.190)	(0.190)
Occupation = 93			-0.428***		-0.507*	-0.501*
			(0.0954)		(0.288)	(0.291)
Sex of twin = o,				-	-	-
Δ wealth						-0.0148
						(0.0154)
Constant	0.422***	0.374***	0.743***	0.491***	0.837***	0.835***
	(0.00885)	(0.0305)	(0.0665)	(0.0944)	(0.217)	(0.218)
Observations	2,303	2,254	2,151	2,254	2,151	2,151
R-squared	0.074	0.079	0.111	0.791	0.805	0.806

Robust standard errors in parentheses
*** p<0.01, ** p<0.05, * p<0.1

Table B.6:
VIF-scores, model 5

Outcome	Free mkt.	Leftright	Party	Redist.	Prop. tax
Wealth	1.17	1.16	1.17		
Fin. wealth				1.06	
Real wealth					1.13
Income	1.57	1.55	1.58	1.54	1.54
Education	2.12	2.13	2.14	2.09	2.11
Children = 1	1.02	1.02	1.03	1.02	1.02
Children = 2	1.02	1.03	1.03	1.03	1.02
Children = 3	1.01	1.01	1.01	1.01	1.01
Occupation = 11	1.31	1.31	1.39	1.34	1.31
Occupation = 12	11.00	11.47	13.47	12.23	11.37
Occupation = 13	4.60	4.61	5.01	5.11	4.99
Occupation = 21	8.45	8.54	9.81	8.85	8.61
Occupation = 22	6.73	6.83	7.81	7.05	6.73
Occupation = 23	13.53	14.00	16.08	15.11	13.99
Occupation = 24	14.55	15.02	18.08	16.16	15.00
Occupation = 31	11.16	11.63	12.95	11.79	11.52
Occupation = 32	8.89	9.55	10.84	9.57	9.08
Occupation = 33	6.38	7.24	8.30	7.29	6.95
Occupation = 34	21.27	21.92	25.11	23.31	21.89
Occupation = 41	18.54	19.26	21.12	20.71	18.91
Occupation = 42	4.85	4.85	5.40	5.27	4.85
Occupation = 51	29.23	30.57	34.38	32.42	30.60
Occupation = 52	6.76	6.77	7.66	7.17	6.76
Occupation = 61	3.23	3.33	3.75	3.88	2.88
Occupation = 71	10.55	10.94	12.36	11.16	10.84
Occupation = 72	6.79	6.79	6.83	7.09	6.88
Occupation = 73	1.82	1.82	2.02	1.80	1.82
Occupation = 74	1.73	1.83	1.77	1.57	1.83
Occupation = 81	2.43	2.43	2.65	2.59	2.33
Occupation = 82	8.19	8.40	9.67	9.07	8.78
Occupation = 83	7.37	7.66	7.80	8.17	7.76
Occupation = 91	9.37	9.38	9.78	9.73	9.48
Occupation = 93	2.26	2.26	2.44	2.51	2.26

1 Appendix C: additional test of H1

Table C.1:
Free market preferences

VARIABLES	(1)<br>Naive	(2)<br>Naive	(3)<br>Naive	(4)<br>Within	(5)<br>Within	(6)<br>Within
Financial wealth, MKr	0.0351**	0.0397**	0.0393**	0.0101	0.00878	0.00553
	(0.0152)	(0.0157)	(0.0162)	(0.0381)	(0.0435)	(0.0439)
Sex of twin		0.0386***	0.0364***			
		(0.00910)	(0.0109)			
Work income		0.0580**	0.0813***	0.0235	0.0293	0.0282
		(0.0258)	(0.0299)	(0.0666)	(0.0767)	(0.0766)
Education years		-0.00598***	-0.00537**	-0.00174	-0.00297	-0.00305
		(0.00177)	(0.00222)	(0.00613)	(0.00683)	(0.00685)
Children under 18 = 1		-0.0182	-0.0164	0.0208	0.0289	0.0280
		(0.0185)	(0.0195)	(0.0393)	(0.0440)	(0.0440)
Children under 18 = 2		-0.0117	-0.0180	-0.0496	-0.0550	-0.0571
		(0.0299)	(0.0300)	(0.0414)	(0.0445)	(0.0452)
Children under 18 = 3		0.0389	0.0572	0.354***	0.388***	0.387***
		(0.112)	(0.118)	(0.0586)	(0.0889)	(0.0896)
Occupation = 11			-0.0776		-0.107	-0.109
			(0.130)		(0.124)	(0.124)
Occupation = 12			-0.0552		0.00937	0.00821
			(0.0419)		(0.0904)	(0.0905)
Occupation = 13			-0.0366		0.0301	0.0260
			(0.0464)		(0.100)	(0.101)
Occupation = 21			-0.102**		-0.0352	-0.0358
			(0.0437)		(0.0990)	(0.0992)
Occupation = 22			-0.0475		0.0145	0.0140
			(0.0466)		(0.102)	(0.102)
Occupation = 23			-0.0790*		0.0124	0.0116
			(0.0425)		(0.0928)	(0.0929)
Occupation = 24			-0.108**		-0.00499	-0.00552
			(0.0422)		(0.0906)	(0.0906)
Occupation = 31			-0.108**		-0.00269	-0.00393
			(0.0424)		(0.0954)	(0.0954)
Occupation = 32			-0.0836*		0.0214	0.0200
			(0.0444)		(0.0965)	(0.0967)
Occupation = 33			-0.111**		-0.00123	-0.00253
			(0.0444)		(0.109)	(0.110)
Occupation = 34			-0.0786*		-0.0170	-0.0185
			(0.0410)		(0.0865)	(0.0868)
Occupation = 41			-0.0624		-0.0167	-0.0183
			(0.0414)		(0.0902)	(0.0905)
Occupation = 42			0.0289		0.0439	0.0440
			(0.0481)		(0.111)	(0.111)
Occupation = 51			-0.0711*		0.0161	0.0151
			(0.0406)		(0.0904)	(0.0905)
Occupation = 52			-0.0377		0.0450	0.0443
			(0.0471)		(0.108)	(0.108)
Occupation = 61			-0.00167		0.0852	0.0802
			(0.0630)		(0.120)	(0.121)
Occupation = 71			-0.0897**		-0.0293	-0.0295
			(0.0444)		(0.0978)	(0.0980)
Occupation = 72			-0.126***		-0.0675	-0.0677
			(0.0464)		(0.101)	(0.101)
Occupation = 73			-0.0669		0.0155	0.0140
			(0.0517)		(0.121)	(0.121)
Occupation = 74			0.0636		0.0924	0.0947
			(0.0818)		(0.127)	(0.128)
Occupation = 81			-0.157***		-0.0662	-0.0679
			(0.0523)		(0.129)	(0.129)
Occupation = 82			-0.113**		0.00940	0.00757
			(0.0451)		(0.101)	(0.102)
Occupation = 83			-0.0765		-0.0245	-0.0256
			(0.0481)		(0.118)	(0.118)
Occupation = 91			-0.0767*		-0.0328	-0.0330
			(0.0440)		(0.0976)	(0.0977)
Occupation = 93			-0.114**		-0.106	-0.109
			(0.0555)		(0.146)	(0.146)
Sex of twin = o,				-	-	-
Δ wealth						0.00387
						(0.00879)
Observations	2,218	2,170	2,068	2,170	2,068	2,068
R-squared	0.003	0.018	0.049	0.777	0.787	0.788
Controls	No	Yes	Yes	Yes	Yes	Yes
Occupation FE	No	No	Yes	No	Yes	Yes

Robust standard errors in parentheses
*** p<0.01, ** p<0.05, * p<0.1

Appendix D: additional outcomes

These are results for outcomes constructed from the first five principal components of the preference data, as previously identified by Oskarsson et al. (2014). The dimensions contain the following questions (level of agreement coded according to sign):

Dimension 1

- Decrease the public sector (+)
- Lower taxes (+)
- Privatize public enterprises (+)
- Privatize healthcare (+)
- Support free schools (+)
- Give companies more freedom (+)

Dimension 2

- Increase economic support to rural areas (+)
- Introduce 6-hour work day for all employees (+)

Dimension 3

- Introduce much harder punishment for criminals (+)
- Increase labor immigration to Sweden (-)
- Introduce a language test to become a Swedish citizen (+)
- Decrease foreign aid (+)
- Accept fewer refugees (+)
- Increase economic support for immigrant culture (-)

Dimension 4

- Invest more in preventing environmental degradation (+)
- Decrease carbon dioxide emissions (+)

Dimension 5

- Sweden should leave the EU (+)
- Sweden should introduce the euro (-)
- Sweden should become a member of NATO (-)

Table D.1:
Dimension 1

VARIABLES	(1) Naive	(2) Naive	(3) Within
Wealth, MKr	0.0309***	0.0287***	0.00854
	(0.00483)	(0.00525)	(0.0156)
Observations	2,201	2,052	2,052
R-squared	0.021	0.055	0.791
Controls	No	Yes	Yes
Occupation FE	No	Yes	Yes

Robust standard errors in parentheses
*** p<0.01, ** p<0.05, * p<0.1

Table D.2:
Dimension 2

VARIABLES	(1) Naive	(2) Naive	(3) Within
Wealth, MKr	-0.0730***	-0.0466***	-0.0281*
	(0.00616)	(0.00641)	(0.0163)
Observations	2,298	2,142	2,142
R-squared	0.081	0.148	0.820
Controls	No	Yes	Yes
Occupation FE	No	Yes	Yes

Robust standard errors in parentheses
*** p<0.01, ** p<0.05, * p<0.1

Table D.3:
Dimension 3

VARIABLES	(1) Naive	(2) Naive	(3) Within
Wealth, MKr	-0.0145***	0.00758	-0.00429
	(0.00508)	(0.00517)	(0.0132)
Observations	2,255	2,107	2,107
R-squared	0.004	0.173	0.858
Controls	No	Yes	Yes
Occupation FE	No	Yes	Yes

Robust standard errors in parentheses
*** p<0.01, ** p<0.05, * p<0.1

Table D.4:
Dimension 4

VARIABLES	(1) Naive	(2) Naive	(3) Within
Wealth, MKr	-0.00828*	-0.00619	-0.0197
	(0.00425)	(0.00459)	(0.0157)
Observations	2,293	2,139	2,139
R-squared	0.002	0.039	0.748
Controls	No	Yes	Yes
Occupation FE	No	Yes	Yes

Robust standard errors in parentheses
*** p<0.01, ** p<0.05, * p<0.1

Table D.5:
Dimension 5

VARIABLES	(1) Naive	(2) Naive	(3) Within
Wealth, MKr	-0.0556***	-0.0321***	-0.0201
	(0.00587)	(0.00656)	(0.0192)
Observations	2,256	2,103	2,103
R-squared	0.042	0.092	0.802
Controls	No	Yes	Yes
Occupation FE	No	Yes	Yes

Robust standard errors in parentheses
*** p<0.01, ** p<0.05, * p<0.1

Appendix E: population comparison

Table E.1 contains a comparison on some key variables where we have data both for the twin sample and the general population. The twins are slightly more educated and have higher incomes, but the differences are fairly negligible. The twin sample also contains a larger proportion of women than would be expected by random chance.

Table E.1:
Descriptive comparison

Sample	Variable	Mean	SD	Min	Max	N
MZ twins						
	Birth year	1950	4.7	1943	1958	2,368
	Education	12.1	2.6	7	19	2,368
	Female	.57	.49	0	1	2,368
	Work income	248832	129638	0	697910	2,309
Population						
	Birth year	1950.2	4.6	1943	1958	2,194,904
	Education	11.6	2.7	7	19	1,886,525
	Female	.49	.50	0	1	2,194,904
	Work income	214228	135162	0	666650	1,823,563

Furthermore, table E.2 contains a comparison between the self-reported party choice in the 2006 national election, and the actual election outcome that year. As we can see, they align very well, with a minor difference for the moderates (30 vs 26 per cent) and the Sweden democrats (1.3 vs 2.9 per cent).

Table E.2:
Party percentages, national election 2006

Party	MZ sample	Election result
Left party	5.03	5.85
Social democrats	35.20	34.99
Green party	6.51	5.24
Center party	7.71	7.88
People's party	5.87	7.54
Christian democrats	7.94	6.59
Moderates	30.44	26.23
Sweden democrats	1.29	2.93
